# Synthesis, Structure and Antibacterial Activity of a Neutral Tin(II) Iodide Complex with 1,2-bis[(4-Methoxyphenyl)imino]acenaphthene

**DOI:** 10.3390/molecules31203676

**Published:** 2026-10-14

**Authors:** Anna A. Tychinina, Ivan V. Smolyaninov, Natalia V. Gogoleva, Anton N. Lukoyanov, Dmitriy S. Yambulatov, Mikhail A. Kiskin

**Affiliations:** 1 Kurnakov Institute of General and Inorganic Chemistry, Russian Academy of Sciences, 31 Leninsky prosp., 119991 Moscow, Russia; anatychinina326@gmail.com (A.A.T.); gogolevanv@inbox.ru (N.V.G.); m_kiskin@mail.ru (M.A.K.); 2 D. Mendeleev University of Chemical Technology of Russia, 9 Miusskaya Square, 125047 Moscow, Russia; 3 Razuvaev Institute of Organometallic Chemistry, Russian Academy of Sciences, 49 Tropinina Street, 603137 Nizhny Novgorod, Russia

**Keywords:** tin(II), tin iodide, bis(imino)acenaphthene, redox-active ligands, coordination compounds, X-ray diffraction, antibacterial activity

## Abstract

The reaction of tin(II) iodide with 1,2-bis[(4-methoxyphenyl)imino]acenaphthene (4-MeO-BIAN) in anhydrous tetrahydrofuran afforded the neutral complex [(4-MeO-BIAN)SnI(μ-I)]_2_ (**1**). A single-phase sample of **1** was isolated and characterized by powder X-ray diffraction and IR spectroscopy. The crystal structure of **1** was determined by single-crystal X-ray diffraction: a centrosymmetric dimeric structure with an Sn_2_I_2_ core formed by two μ_2_-bridging iodide ligands; each Sn(II) center is additionally coordinated by a bidentate 4-MeO-BIAN ligand and one terminal iodide ligand. The antibacterial activity of **1** was evaluated in vitro against clinically significant Gram-positive (*Staphylococcus aureus*, *Enterococcus faecium*, *Enterococcus faecalis*) and Gram-negative (*Escherichia coli*) bacteria. Complex **1** exhibited moderate antibacterial activity against all tested strains, with MIC values of 40–312.5 µg/mL and MBC values of 62.5–312.5 µg/mL. The lowest MIC values, close to those of meropenem (MIC = 32 µg/mL), were obtained against *Staphylococcus aureus* (MIC = 40 µg/mL).

## 1. Introduction

Redox-active ligands occupy a special place in modern coordination and bioinorganic chemistry. Unlike “classical” ligands, they are capable of participating in redox processes as well as the central metal atom, reversibly changing their oxidation state and acting as electron reservoirs [1,2,3,4,5,6,7]. The redox-active behavior of ligands is widespread in natural systems: molecules such as *o*-quinones, pterins, flavins, and tetrapyrroles function as redox cofactors in the active centers of metalloenzymes containing iron, copper, manganese, and molybdenum [8]. Metal complexes with redox-active ligands are of considerable interest as potential drugs: they can be activated through ligand substitution reactions or redox processes and can simultaneously affect multiple biological targets [9,10,11]. This ability underlies the high potential of such compounds as antibacterial and antitumor agents [11,12,13].

Among redox-active ligands, particular attention should be paid to acenaphthene derivatives—bis(arylimino)acenaphthenes (Ar-BIAN) and mono(arylimino)acenaphthenones (Ar-MIAN) [14,15,16]. Due to the rigid conjugated π-system of the acenaphthene core and two imine groups, these ligands possess high electron affinity and can exist in several oxidation states: neutral, radical-anion, and dianionic [17]. The coordination chemistry of BIAN and MIAN with transition metals is actively developing: complexes of rhodium [18,19,20], vanadium [21], copper [22], palladium [23,24], and platinum [17] have been obtained and characterized with SC-XRD. Au(I) complexes with BIAN and MIAN ligands also have been structurally characterized by X-ray diffraction, and preliminary biological activity tests have been performed for all complexes [25].

Although tin is not an essential metal, its compounds have long attracted the attention of researchers as potential biologically active agents [26]. Tin complexes exhibit a wide range of pharmacological effects, including antibacterial, antifungal, antioxidant, and antitumor activities [26,27,28,29,30,31,32]. The coordination of organic ligands to Sn(II) leads to a significant enhancement of the biological effect compared to the free ligands, which is attributed to an increase in the lipophilicity of the complexes and the facilitation of their penetration through microbial cell membranes [33]. Thus, tin(II) complexes represent a promising platform for the development of new biologically active compounds [26,28,34,35].

Despite the interest in redox-active acenaphthene derivatives (BIAN, MIAN) and the bioactive properties of tin(II) compounds, their complexes to the best of our knowledge remain poorly studied. The available works on tin with redox-active ligands mainly concern tin(IV) or focus on catalytic and electrochemical properties [36,37,38]. The aim of this work is the synthesis, structural and electrochemical characterization of the neutral complex [(4-MeO-BIAN)SnI(μ-I)]_2_, as well as the evaluation of its antibacterial activity. The structure and purity of the obtained compound were characterized by single-crystal and powder X-ray diffraction, IR spectroscopy, and elemental analysis.

## 2. Results and Discussion

### 2.1. Synthesis and Characterization of ***1***

The reaction of equimolar amounts of tin(II) iodide and the 4-MeO-BIAN ligand in anhydrous THF afforded dark brown crystals of the complex [(4-MeO-BIAN)SnI(μ-I)]_2_ (**1**) in 84% yield.

**Scheme 1:**
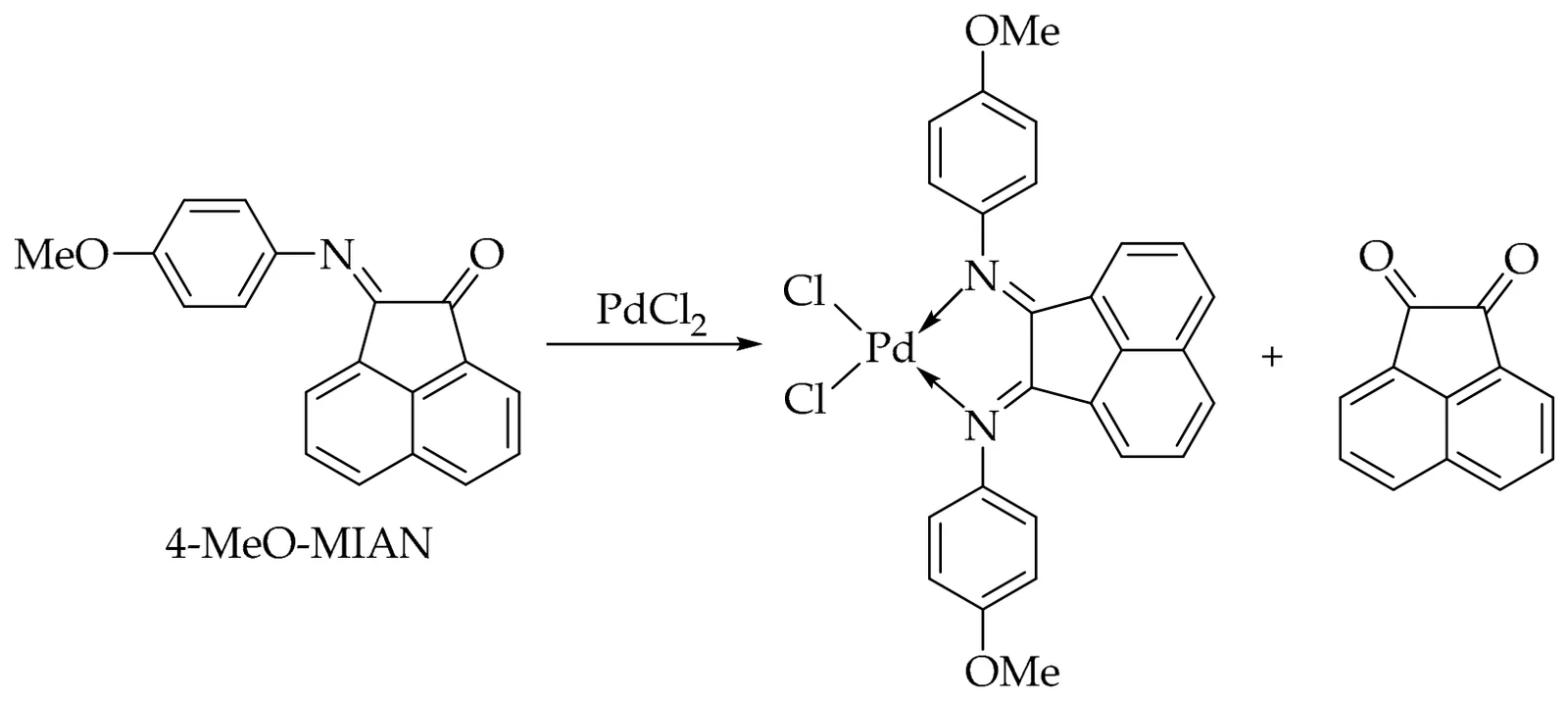
Dimerization of 4-MeO-MIAN into the corresponding BIAN in the reaction with PdCl_2_.

The obtained results demonstrate that the use of 4-MeO-MIAN in the reaction with SnI_2_ leads to the formation of the BIAN-containing complex **1**. Similar MIAN-to-BIAN transformations have previously been observed for other metal systems and were attributed to disproportionation of two MIAN molecules with the formation of the corresponding BIAN derivative and acenaphthenequinone: the reaction of PdCl_2_ with 4-methoxyphenyl-substituted MIAN in dichloromethane is accompanied by a rearrangement of the ligand structure, resulting in the formation of the complex [(4-MeO-BIAN)PdCl_2_] containing the corresponding BIAN ligand; two MIAN molecules undergo “coupling” to form a diimine BIAN structure in the coordination sphere of the metal (Scheme 1) [21,39]. Our previous studies demonstrated that 4-MeO-MIAN retains its monoiminoacenaphthenone structure upon coordination to a heterometallic Co(II)–Li(I) system [40], whereas its transformation into the corresponding BIAN derivative has been observed for other metal ions [39]. These contrasting coordination behaviors prompted us to investigate the interaction of this particular redox-active ligand system with Sn(II) and to determine whether coordination or ligand rearrangement would occur under these conditions.

However, isolation and identification of the organic side products were not the aim of the present study, and acenaphthenequinone was not unambiguously identified in the reaction mixture. Therefore, although the overall ligand transformation appears similar to that reported previously, the available experimental data do not allow us to establish whether it proceeds through exactly the same reaction pathway.

**Scheme 2:**
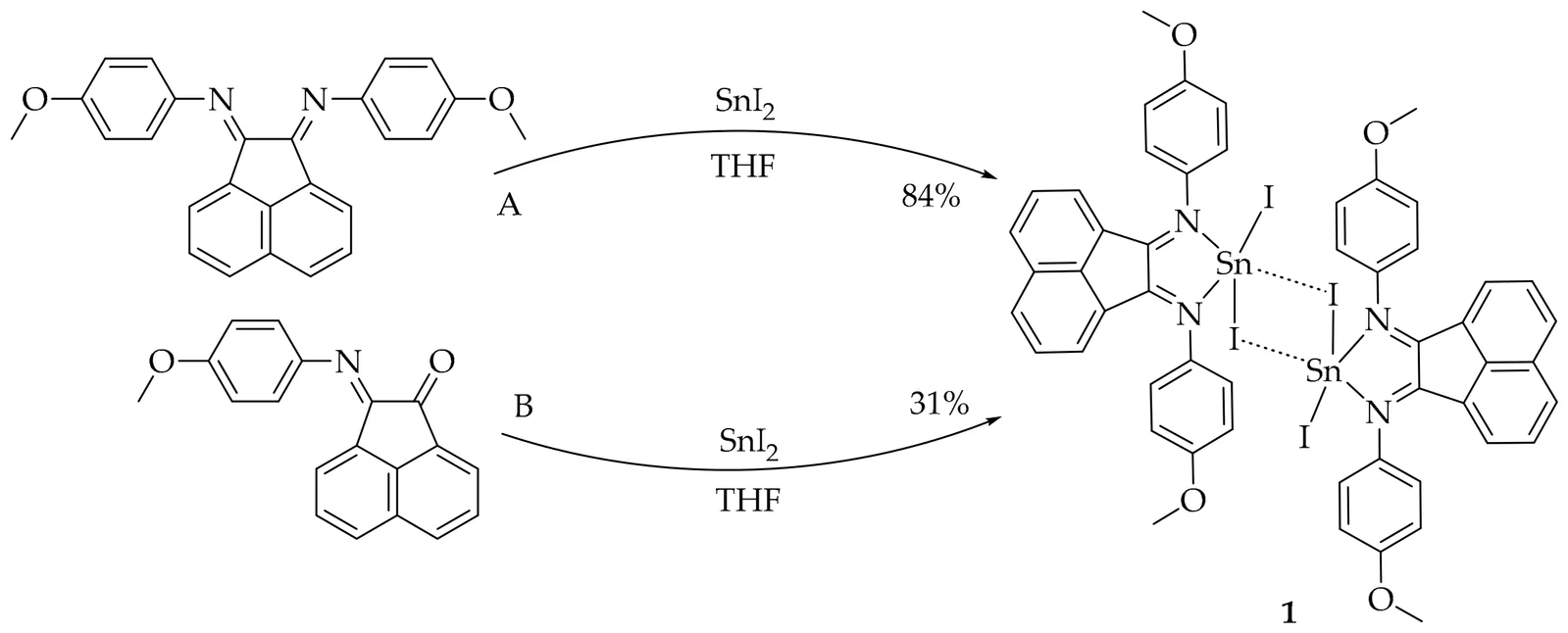
Synthesis of complex **1**.

The obtained results indicate that a similar transformation also occurs in the presence of Sn^2+^ ions, leading to the BIAN complex (Scheme 2).

At the same time, as was mentioned earlier, when the MIAN ligand (4-MeO-MIAN) was used in the cobalt(II)–lithium(I) complex [Co_2_Li_2_(piv)_6_(L)_2_] (piv is an anion of trimethylacetic acid), no rearrangement of the ligand structure was observed, and the MIAN ligand retained its original monoiminoacenaphthenone structure, coordinating through nitrogen and oxygen atoms [40]. Thus, the ability of MIAN ligands to undergo rearrangement depends on the nature of the metal and solvent, the Lewis acidity and the reaction conditions.

The presence of the coordinated 4-MeO-BIAN ligand in **1** was confirmed by IR spectroscopy (see Supplementary Materials, Figure S1). The experimental data confirm the presence of characteristic bands corresponding to C=N stretching vibrations (1697, 1650, 1596, and 1577 cm^−1^) and C–O–C vibrations of the aromatic ether (1242 and 1018 cm^−1^). In contrast to complexes containing MIAN ligands, the spectrum of complex **1** does not show the intense band of the carbonyl group C=O stretching vibrations in the region of ~1720 cm^−1^, which indicates a rearrangement of the ligand structure with the formation of the BIAN derivative, in agreement with the X-ray diffraction data.

### 2.2. Crystal Structures of ***1***

**Figure 1:**
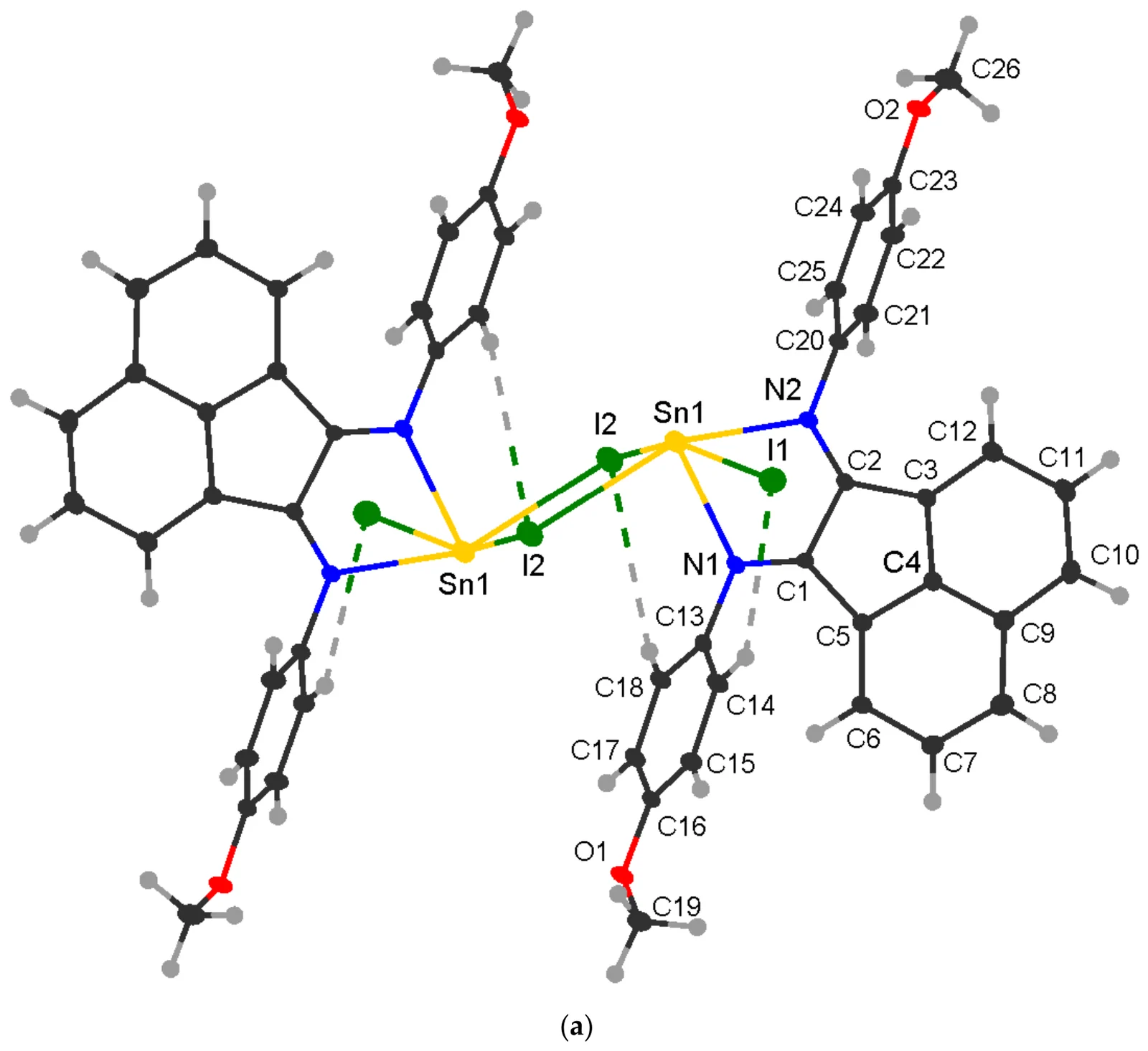

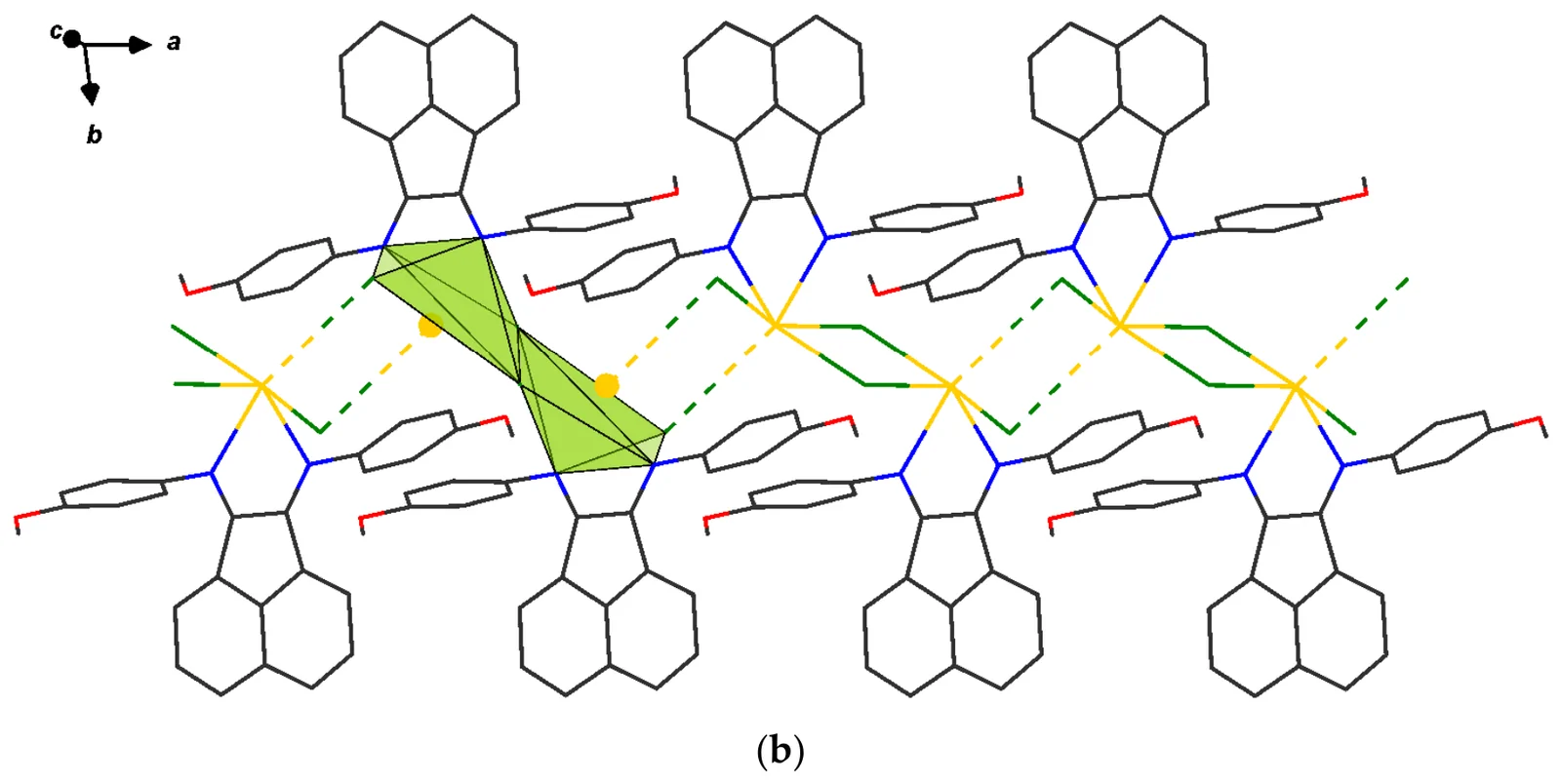
Molecular structure of complex **1** ((**a**), ellipsoids at 30% probability; intramolecular C–H···I contacts are shown by dashed line); fragment of the crystal packing and geometry of coordination polyhedron SnN_2_I_3_ ((**b**), hydrogen atoms are omitted; intramolecular Sn1···I1 contacts are shown as dashed lines).

According to single-crystal X-ray diffraction data, complex **1** has a dimeric structure (Figure 1a), in which two tin atoms are symmetrically linked to each other by two μ_2_-bridging I2 atoms (Sn–I 3.0908(4), 3.3316(11) Å, I–Sn–I 90.31(3)°, Sn–I–Sn 90.28(16)°; an inversion center is located at the center of the Sn_2_I_2_ rectangle). The metal atom completes its coordination sphere with two nitrogen atoms of the chelating 4-MeO-BIAN molecule (Sn–N 2.428(3), 2.511(3) Å, N–Sn–N 67.73(11)°) and a terminal iodine atom (Sn–I1 2.9631(4) Å, I1–Sn–I2 93.084(10)°), forming a square pyramid (SnN_2_I_3_) with the base formed by N2, I1, and two I2 atoms, from the plane of which the tin atom deviates by 0.5591(13) Å, with the apex formed by the N1 atom (τ_5_ = 0.02, Figure 1b), or a vacant octahedron (S_Q_ = 3.760), in which the lone electron pair on the tin atom is directed toward the I1 atom of the neighboring dimer (Sn···I 3.7570(4) Å) with the formation of a supramolecular chain along the 0*a* axis (Figure 1b).

In the coordinated 4-MeO-BIAN molecule, the iminoacenaphthene fragment (C1–N1 1.282(5) Å, C2–N2 1.272(5) Å, C1–C2 1.508(5) Å, C–C_range_ 1.377(7)–1.484(5) Å) is planar, with a root-mean-square deviation of the atoms from the mean plane of 0.061 Å. The phenyl fragments are rotated relative to the BIAN core, with the C–N–C–C dihedral angles being 51.9(6)° and 89.8(5)°. The larger rotation of the aromatic substituent is due to the formation of an intramolecular contact between the hydrogen atom at the 2-position of the phenyl ring and I2 atom (Figure 1a, Table 1). In the crystal, the molecules of the complex additionally interact with each other through non-covalent C–H···O, C–H···I, and C–H···π interactions, forming a three-dimensional network (Table 2).

The experimental powder X-ray diffraction pattern of the bulk sample is in good agreement with that calculated from the single-crystal X-ray diffraction data, confirming that complex **1** represents the predominant crystalline phase of the isolated material (Figure S3).

### 2.3. Electrochemical Studies

**Figure 2:**
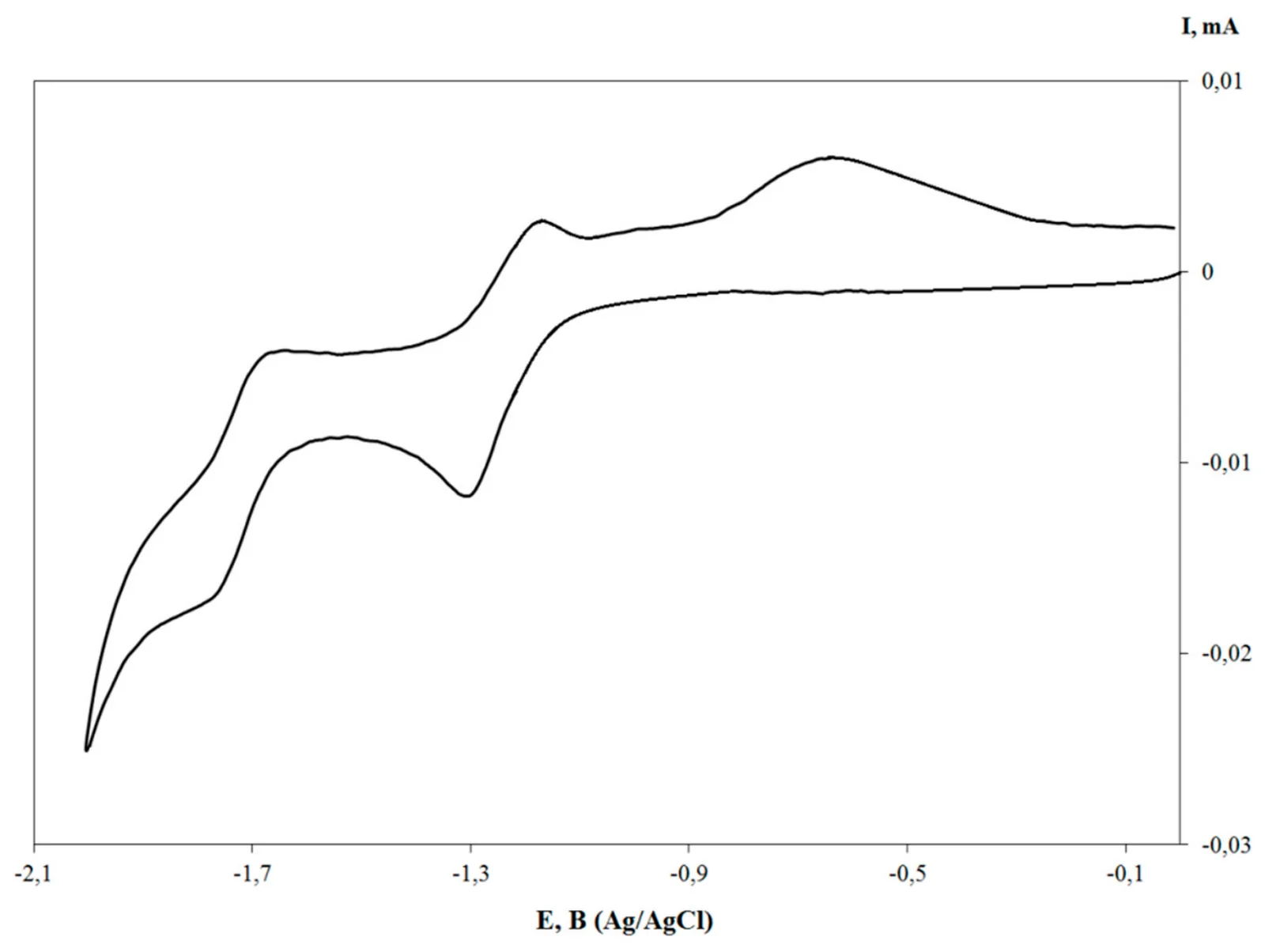
The CV reduction curve of complex **1** was recorded in the potential range from 0.0 to −2.0 V (CH_2_Cl_2_, glassy carbon electrode, C(I) = 0.0015 M, 0.15 M nBu_4_NClO_4_, scan rate 0.2 V/s, Ag/AgCl/KCl).

The electrochemical behavior of complex **1** was studied by cyclic voltammetry (CV) in dichloromethane at a glassy carbon electrode (Figure 2).

**Scheme 3:**
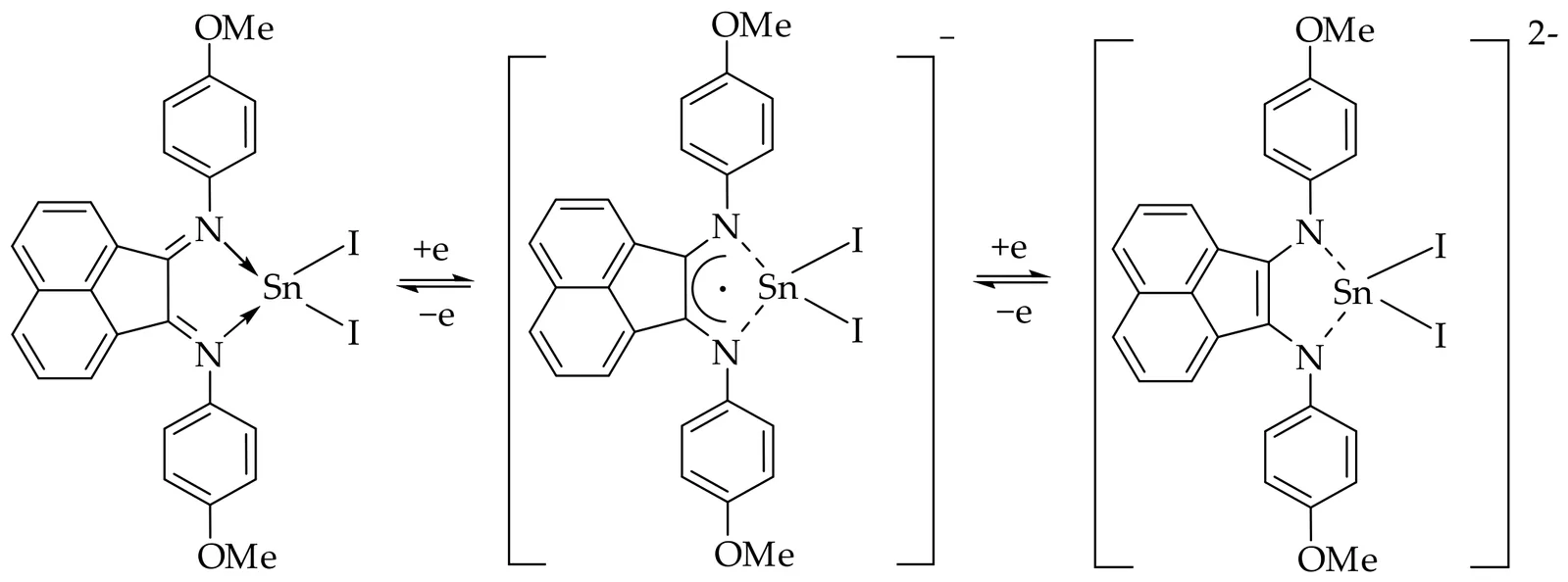
Reduction of complex **1** with the formation of the mono- and dianionic species.

For complex **1**, two quasi-reversible reduction peaks are observed at E^red1^_1/2_ = −1.26 V and E^red12^_1/2_ = −1.73 V. The current ratios for the first and second cathodic steps are 0.65 and 0.62, respectively, indicating a relative stability of the electrogenerated intermediates. The electron transfer is followed by a fast chemical step, which leads to a decrease in the current ratio and the appearance of a re-oxidation peak of the product of the chemical step at 0.65 V on the reverse scan of the voltammogram. The observed CV peaks can be attributed to the formation of mono- and dianionic complexes containing the one- and two-electron reduced forms of the redox-active ligand (Scheme 3, **1** as monomer for clarity).

**Figure 3:**
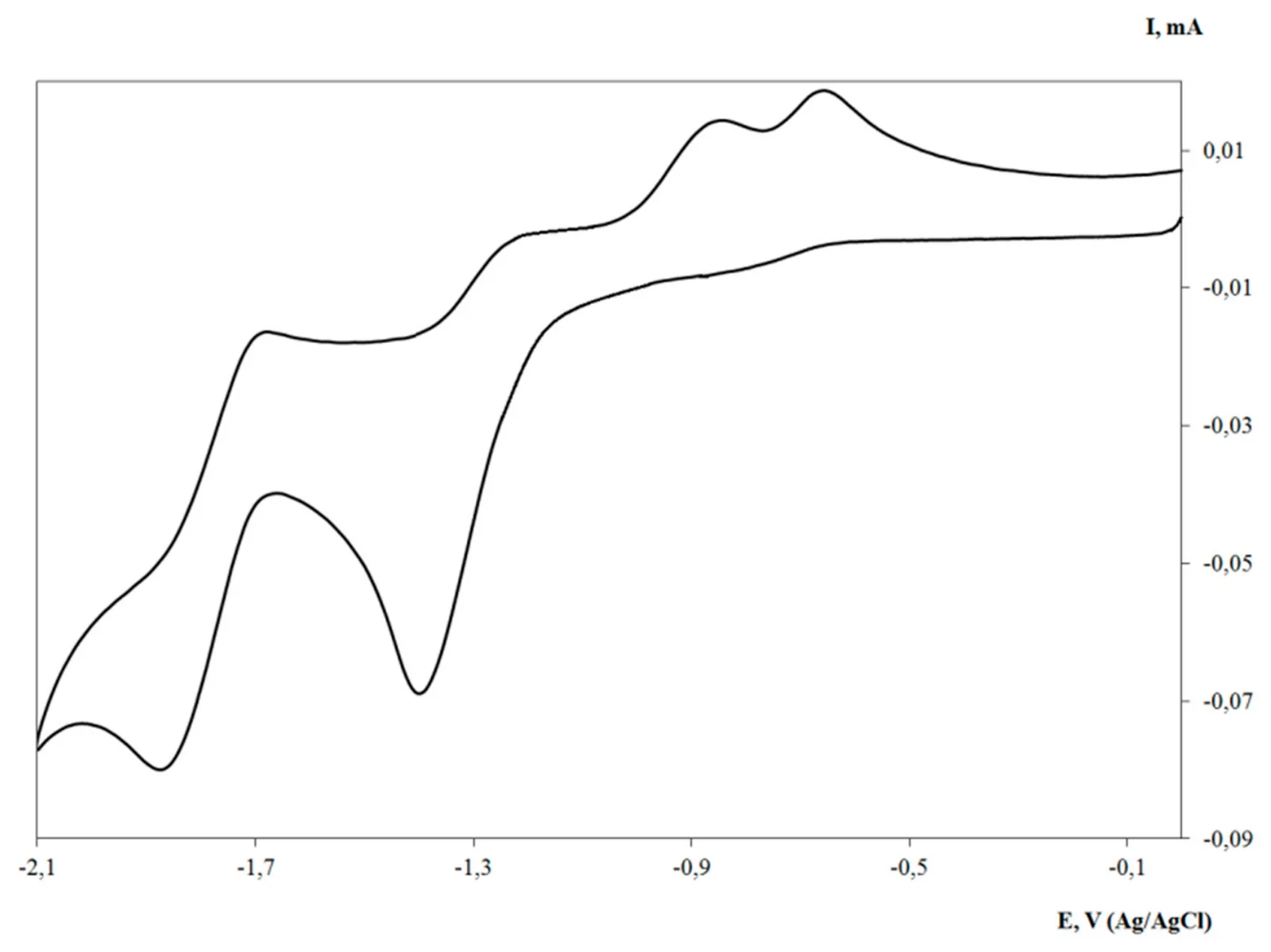
The CV reduction curve of free BIAN was recorded in the potential range from 0.0 to −2.1 V (CH_2_Cl_2_, glassy carbon electrode, C(I) = 0.0035 M, 0.15 M nBu_4_NClO_4_, scan rate 0.2 V/s, Ag/AgCl/KCl).

In contrast to the previously studied zinc(II) complexes with redox-active ligands [41,42,43], the interaction of the BIAN ligand with tin(II) iodide, which is a weak Lewis acid, results in a slight anodic shift in the reduction potentials of the redox-active ligand by 0.07–0.08 V compared to the free BIAN (Figure 3).

Under similar experimental conditions, the ligand undergoes electroreduction in two quasi-reversible one-electron steps at E^red1^_1/2_ = −1.34 V and E^red12^_1/2_ = −1.80 V. The current ratios are 0.44 and 0.78 for the first and second redox transitions, respectively. For the free BIAN ligand, the reverse scan of the CV curves shows re-oxidation peaks at −0.85 and −0.65 V. The electrogenerated mono- and dianionic forms exhibit increased basicity and are capable of protonation. The re-oxidation peak observed in the CV of complex **1** at −0.65 V can be attributed to the product of decoordination of the doubly reduced protonated form of the ligand. In the anodic region, an irreversible oxidation peak is observed for complex **1** at 0.93 V, presumably corresponding to the oxidation of the Sn–I bond. A similar peak at 0.73–0.86 V was previously reported for zinc iodide and its complexes with redox-active ligands.

### 2.4. Antibacterial Activity

The antibacterial activity of the synthesized complex **1**, the related compound 4-MeO-MIAN, and the starting SnI_2_ was evaluated against clinically significant strains of Gram-positive and Gram-negative bacteria (Table 3 and Table 4). The parent tin salt exhibited pronounced bacteriostatic activity against *S. aureus* and *E. faecalis* ATCC 51299, with MIC values ranging from 40 to 62.5 µg/mL. At the same time, low antibacterial activity was observed for the strains *E. faecium* 1165, *E. faecium* 3994, and *E. faecalis* 3576. For the first five strains, the MIC values of complex **1** are comparable to those of the parent tin salt. Selectivity of action was noted for the strains *S. aureus* O1 and R1 (clinical isolates resistant to meropenem), as well as *E. faecium* 1165 and *E. faecalis* 3576 (78 µg/mL), surpassing the data for meropenem. The antibacterial activity of complex **1** is comparable to that of SnI_2_ for several of the tested strains, while differences are observed for some clinical isolates. Since the antibacterial activity of free 4-MeO-BIAN was not determined in the present study, the individual contribution of the coordinated BIAN ligand to the activity of complex **1** cannot be unambiguously assessed.

The initial tin salt, complex **1**, and 4-MeO-MIAN do not exhibit pronounced antibacterial activity against the Gram-negative bacterium *E. coli* ATCC 25922: the MIC values ranged from 156 to 312.5 µg/mL.

Comparison of the minimum inhibitory concentration (MIC) and minimum bactericidal concentration (MBC) for the studied compounds reveals activity against Gram-positive cocci. All three compounds exhibit moderate antibacterial activity against *S. aureus* strains (including GISA) and enterococci. The MBC/MIC ratio is a characteristic of the type of action of a substance. For meropenem, the MBC equals the MIC in all cases, confirming its bactericidal effect. For the studied compounds, the MBC/MIC ratio in most cases ranges from 1 to 5, which allows them to be classified as predominantly bactericidal agents, although less pronounced than meropenem. In the case of SnI_2_, for staphylococci and some enterococci (e.g., *E. faecalis* 3576), the MBC/MIC ratio is 2, indicating bactericidal action. However, for *E. faecium* ATCC 29212 and strains 1165 and 3994, this ratio increases to 5–7.8, meaning that the effect becomes bacteriostatic at the MIC, and cell death occurs only at concentrations 5–8 times higher than the MIC. For *E. coli*, the MBC of SnI_2_ is 625 µg/mL (MIC 156, ratio 4), indicating very weak bactericidal activity against this Gram-negative bacterium. The effect of the 4-MeO-MIAN ligand on bacteria is more diverse. Against *S. aureus* GISA and strain R1, the MBC/MIC ratio is 5 (bacteriostatic profile), while against *E. faecium* ATCC 29212 and *E. faecium* 1165, this parameter indicates bactericidal action with a ratio of 1.6–2. Against *E. coli*, with MBC = 312.5 µg/mL, the compound is moderately bactericidal.

Complex **1** exhibits the most stable bactericidal profile. For most strains (all three *S. aureus* strains, *E. faecalis* ATCC 51299, *E. faecium* 3994, and *E. faecalis* 3576), the MBC/MIC ratio is 1–2, meaning that the bactericidal effect is achieved already at a concentration equal to or twice the MIC. Exceptions are *E. faecium* ATCC 29212 and *E. faecium* 1165, for which the bactericidal effect is noticeably weaker. Against *E. coli*, complex **1** is almost inactive, indicating a complete absence of bactericidal activity against Gram-negative microflora in the concentration range studied. The inefficacy against *E. coli* is typical for substances with low ability to penetrate the outer membrane of Gram-negative bacteria, which is associated with the presence of an additional outer membrane that hinders the penetration of the complex into the cell.

The predominance of MBC values exceeding the MIC by no more than 4 times indicates that the studied compounds are not purely bacteriostatic agents but are capable of causing cell death at concentrations close to the inhibitory ones. Complex **1** is distinguished by its bactericidal activity, which is achieved already at the MIC (for many strains) or at a twofold excess.

In summary, the studied compounds exhibit moderate but stable activity against Gram-positive cocci, including strains with reduced susceptibility to meropenem. Based on the MBC/MIC ratio, they range from strictly bactericidal (complex **1**) to moderately bacteriostatic (in certain cases for SnI_2_ and 4-MeO-MIAN). The most predictable bactericidal effect is demonstrated by compound **1**, which combines a redox-active ligand and a tin atom.

Further studies including direct comparison with the corresponding free BIAN ligand are required to clarify the effect of complex formation on the antibacterial activity.

It should be noted that complex **1** and SnI_2_ exhibit identical or similar MIC values against several of the tested strains. The stability and speciation of complex **1** under the conditions of the antibacterial assays have not been investigated in the present study. Therefore, partial dissociation, hydrolysis, or other transformations of the complex in the culture medium cannot be excluded, and the observed antibacterial activity cannot be unequivocally attributed to the intact coordination complex. Further solution-stability and speciation studies under physiologically relevant conditions are required to clarify the nature of the biologically active species.

## 3. Materials and Methods

### 3.1. General Remarks

The synthesis of compound **1** was carried out using a modified Schlenk technique. Photographs of the main synthesis stages are available in the Supplementary Materials (Figure S2). Tetrahydrofuran (pure grade, “Khimmed”) was dried by refluxing over sodium with benzophenone until a blue color appeared, then distilled and stored over sodium with benzophenone; it was condensed immediately prior to synthesis. Tin(II) iodide was synthesized in situ from elemental tin and iodine in tetrahydrofuran in a glass ampoule with a Teflon stopcock under heating. The diimine ligand (4-MeO-BIAN) and its monoimino derivative (4-MeO-MIAN) were obtained according to previously reported procedures [40,44].

IR spectra were recorded in the range of 400–4000 cm^−1^ on a Perkin Elmer Spectrum 65 spectrophotometer (Waltham, MA, USA) equipped with a Quest ATR Accessory (Specac, Orpington, UK) using the attenuated total reflection (ATR) technique. Elemental analysis was performed on an automatic CHNS analyzer EuroEA-3000 (EuroVektor, Pavia, Italy).

Single-crystal X-ray diffraction data of **1** were collected on a Bruker D8 Venture diffractometer equipped with a two-dimensional CCD detector using MoKα radiation (λ = 0.71073 Å) and a graphite monochromator. Absorption was corrected empirically using the SADABS program [45]. The structure was solved using SHELXT [46] incorporated in the OLEX^2^ 1.5 program package [47]. All non-hydrogen atoms were located from difference Fourier maps and refined anisotropically against F^2^_hkl_ using SHELXL 2018/3 [46]. Hydrogen atoms on carbon atoms of the organic ligands were generated geometrically and refined using a riding model. Crystallographic data, experimental parameters at T = 100(2) K, and refinement details are as follows: CCDC No. 2584822, formula C_26_H_20_I_2_N_2_O_2_Sn, M = 764.93 g mol^−1^, triclinic space group *P*–1, *a* = 9.1383(5) Å, *b* = 12.1567(5) Å, *c* = 13.4549(7) Å, α = 64.274(2)°, β = 78.066(2)°, γ = 69.895(2)°, *V* = 1261.58(11) Å^3^, *Z* = 2, *T*_min_*/T*_max_ = 0.4663/0.7467, μ = 3.485 mm^−1^, *d*_calc_ = 2.014 g cm^−3^, *θ*_max_ = 34.02°, *R*_int_ = 0.0274, 14,356 measured reflections, 8391 independent reflections, 7694 reflections with *I* > 2σ(*I*), *GooF* = 1.153, *R*_1_(*I* > 2σ(*I*)) = 0.0536, *wR*_2_(*I* > 2σ(*I*)) = 0.1552, *R*_1_(all) = 0.0562, *wR*_2_(all) = 0.1577, ρ_min_/ρ_max_ = −3.180/1.952 e Å^−3^.

Powder X-ray diffraction data for compound **1** were obtained at 20 °C on a Bruker D8 Advance diffractometer (CuKα, λ = 1.54060 Å, Ni filter, reflection geometry, LynxEye detector). The measurement was performed in the 2θ range of 5–50° with a step of 0.020°. The sample was finely dispersed on a zero-background silicon holder. Rietveld refinement was carried out using TOPAS 4 software [48]. The structure of compound **1** determined by single-crystal X-ray diffraction at low temperature was used as the initial model.

Electrochemical measurements were performed by cyclic voltammetry (CV) using an IPC-pro potentiostat (Russia, LLC STF “Volta”, St. Petersburg, Russia) in a three-electrode cell under an argon atmosphere in dichloromethane. A stationary glassy carbon (GC) electrode with a diameter of 2 mm served as the working electrode, and a platinum plate (S = 18 mm^2^) was used as the auxiliary electrode. The reference electrode was Ag/AgCl/KCl with a waterproof diaphragm. The scan rate was 0.2 V s^−1^. Tetrabutylammonium perchlorate (0.15 M n-Bu_4_NClO_4_) was used as the supporting electrolyte.

The antibacterial activity of the synthesized complex **1**, the related compound 4-MeO-MIAN, and the starting SnI_2_ was studied by the microdilution method in plates against clinically significant strains of Gram-positive and Gram-negative bacteria. The test cultures included reference and clinical strains of *Staphylococcus aureus*, *Enterococcus faecium*, *Enterococcus faecalis*, and *Escherichia coli*. Meropenem was used as the reference antibiotic. Stock solutions of the compounds were prepared in DMSO at a concentration of 5000 µg/mL (500 µg/mL for repeated experiments). The minimum inhibitory concentration (MIC) was determined after 20–24 h of incubation, and the minimum bactericidal concentration (MBC) was determined after 48 h. The MIC and MBC assays were performed in triplicate in two independent experiments. The obtained endpoint values were identical across the replicate measurements; therefore, the corresponding standard deviation was zero.

Antibacterial activity was assessed against bacterial strains *S. aureus* ATCC 29213, *S. aureus* ATCC 6538P, and *E. coli* ATCC 25922 obtained from the American Type Culture Collection (ATCC, Manassas, VA, USA). The minimum inhibitory concentration (MIC, µg/mL) was determined by the broth microdilution method in Mueller–Hinton broth (MHB) according to the Clinical and Laboratory Standards Institute (CLSI) guidelines [49]. The test compounds were dissolved in dimethyl sulfoxide (DMSO) at an initial concentration of 5000 µg/mL (500 µg/mL for repeated experiments), diluted with MHB, and tested in a concentration range from 312.50 to 0.61 µg/mL. The final DMSO concentration was below 12.5% and did not affect microbial growth. Inoculates were adjusted to approximately 5 × 10^5^ CFU/mL. The diluted bacterial cultures in MHB were incubated at 37 °C for 24 h. MIC values were determined visually as the lowest concentration of the test compound resulting in complete inhibition of growth. The minimum bactericidal concentration (MBC) was determined after 48 h. Each assay was performed in triplicate in two independent experiments. DMSO at a concentration of 12.5% was used as a negative control, and meropenem was used as a positive control.

### 3.2. Synthesis of [(4-MeO-BIAN)SnI(μ-I)]_2_ (***1***)

Tin(II) iodide was prepared in situ by the reaction of elemental tin with iodine in anhydrous THF. A detailed synthetic procedure and characterization of the obtained SnI_2_ by powder X-ray diffraction are provided in the Supporting Information (Figure S4).

Method A. A solution of SnI_2_ in THF (0.5 mmol in 15 mL) was prepared in a separate glass ampoule with a Teflon stopcock and transferred into a glass ampoule with a magnetic stir bar. In another glass ampoule, a sample of 4-MeO-BIAN (0.1963 g, 0.5 mmol) was placed and degassed under dynamic vacuum for 20 min. Anhydrous THF (5 mL) was condensed into the ampoule with the ligand, and the resulting solution was then poured into the ampoule with the SnI_2_. The latter was sealed and left to stir at room temperature for 3 h (the color of the reaction mixture was brown). Stirring of the reaction mixture led to the formation of dark red crystals of compound (**1**). The yield was 0.321 g (84%).

Method B. A solution of SnI_2_ in THF (0.5 mmol in 15 mL) was prepared in a separate glass ampoule with a Teflon stopcock and transferred into a glass ampoule. In another glass ampoule, a weight amount of 4-MeO-MIAN (0.1437 g, 0.5 mmol) was placed and degassed under dynamic vacuum for 20 min. Anhydrous THF (5 mL) was condensed into the ampoule with the ligand, and the resulting solution was then poured into the ampoule with the SnI_2_. The latter was sealed and heated in an oil bath at 80 °C for 3 h. Heating of the reaction mixture led to the formation of dark red crystals of compound (**1**). The yield was 0.119 g (31%).

Elemental analysis calculated for C_26_H_20_I_2_N_2_O_2_Sn (%): C, 40.83; H, 2.64; N, 3.66. Found (%): C, 39.77; H, 2.88; N, 3.76.

IR spectrum (ATR; ν, cm^−1^): 3902 w, 3873 w, 3853 w, 3838 w, 3818 w, 3801 w, 3748 w, 3734 w, 3705 w, 3674 w, 3649 w, 3612 w, 3561 w, 2962 w, 2902 w, 2830 w, 1697 w, 1650 w, 1596 w, 1577 w, 1499 m, 1460 w, 1433 w, 1415 w, 1290 w, 1242 s, 1178 m, 1164 m, 1096 s, 1018 vs, 944 w, 853 w, 825 s, 798 vs, 775 s, 716 m, 689 w, 650 w, 594 m, 547 m.

^1^H NMR (300 MHz, DMSO-*d6*) δ, ppm: 8.09 (d, 2H, C(5)H, J = 8.2 Hz), 7.54 (t, 2H, C(4)H, J = 7.6 Hz), 7.11–7.03 (m, 8H, C_6_H_4_), 6.93 (d, 2H, C(3)H, J = 7.2 Hz), 3.83 (s, 6H, OMe).

## 4. Conclusions

Thus, a new tin(II) complex with the redox-active ligand 4-MeO-BIAN has been synthesized and characterized by single-crystal X-ray diffraction, IR spectroscopy, elemental analysis, and powder X-ray diffraction. It was also found that the molecular complex can be formed in the reaction of SnI_2_ with 4-MeO-MIAN as a result of rearrangement at the metal center into the corresponding symmetrical ligand BIAN, but with a lower yield. Based on the obtained structural data, it has been demonstrated that the reaction product represents a binuclear molecule featuring two bridging iodide anions and chelate-bound ligands in their neutral form. Intermolecular Sn···I interactions give rise to the formation of a one-dimensional supramolecular chain. Cyclic voltammetry revealed that the reduction of complex **1** in CH_2_Cl_2_ proceeds in two consecutive quasi-reversible steps, corresponding to the formation of the mono- and dianionic forms of the complex containing the radical-anionic and doubly reduced forms of the redox-active ligand. Coordination of the α-diimine ligand to tin(II) iodide, acting as a weak Lewis acid, results in an anodic shift in the redox transitions by 0.07–0.08 V compared to the free BIAN ligand, indicating a weak influence of the metal center on the redox properties of the ligand. Complex **1** was shown to exhibit antibacterial activity in vitro against clinically significant strains of Gram-positive bacteria, which opens up prospects for further investigation of its biological properties.

## Figures and Tables

**Table 1:** Main parameters of C–H···A contacts in the crystal of complex **1**.

C–H···A	Symmetry Operation	C–H, Å	H···A, Å	C···A, Å	C–H···A, °
C14–H14···I1	–	0.95	3.20	3.957	138
C8–H8···O2	1 + *x*, −1 + *y*, *z*	0.95	2.44	3.356(6)	162
C19–H19B···I1	−*x* + 1, −*y* + 1, −*z* + 1	0.95	3.26	4.164	154
C24–H24···I2	−*x*, −*y* + 2, −*z* + 1	0.98	3.21	3.900	131
C17–H17···I1	1 + *x*, *y*, *z*	0.95	3.00	3.725(5)	134
C26–H26B···O1	−1 + *x*, *y*, 1 + *z*	0.98	2.56	3.538(7)	173
C26–H26C···I2	−1 + *x*, *y*, 1 + *z*	0.98	3.03	3.984(6)	166

**Table 2:** Main parameters of C–H···π contacts in the crystal of complex **1** (Cg_i_ is the centroid of the corresponding six-membered ring; H–Perp is the perpendicular distance from H to the ring plane; γ is the angle between the C–Cg_i_ vector and the normal to the ring plane).

C–H···Cg_i_	Symmetry Operation	H···Cg, Å	H–Perp, Å	γ, °	C–H···Cg, °	C···Cg, Å
C6–H6···Cg_6_(C13–C18)	–	2.59	2.51	14.88	143	3.402(4)
C15–H15···Cg_5_(C4–C9)	1 − *x*, 1 − *y*, 1 − *z*	2.79	2.72	12.29	144	3.598(5)
C19–H19A···Cg_4_(C3C4C9–C12)	1 − *x*, 1 − *y*, 1 − *z*	2.93	2.92	4.53	135	3.695(6)
C26–H26A···Cg_7_(C20–C25)	−*x*, 2 − *y*, 2 − *z*	2.82	2.75	12.87	162	3.764(7)

**Table 3:** Minimum inhibitory concentration (MIC) of the compounds, µg/mL.

Strain	SnI_2_	4-MeO-MIAN	Complex 1	Meropenem
*S. aureus* GISA ATCC 700699	62.5	62.5	**62.5**	32
*S. aureus* O1	40	78	**40**	32
*S. aureus* R1	78	78	**40**	32
*E. faecium* ATCC 29212	40	40	**40**	4
*E. faecalis* ATCC 51299	62.5	62.5	**62.5**	256
*E. faecium* 1165	156	31.2	**78**	128
*E. faecium* 3994	156	62.5	**156**	16
*E. faecalis* 3576	156	156	**78**	256
*E. coli* ATCC 25922	156	156	**312.5**	–

**Table 4:** Minimum bactericidal concentration (MBC) of the compounds, µg/mL.

Strain	SnI_2_	4-MeO-MIAN	Complex 1	Meropenem
*S. aureus* GISA ATCC 700699	62.5	312.5	**156.3**	32
*S. aureus* O1	78	156.3	**78**	32
*S. aureus* R1	78	312.5	**156**	32
*E. faecium* ATCC 29212	312.5	62.5	**62.5**	4
*E. faecalis* ATCC 51299	62.5	62.5	**62.5**	256
*E. faecium* 1165	312.5	62.5	**312.5**	128
*E. faecium* 3994	312.5	312.5	**156**	16
*E. faecalis* 3576	312.5	312.5	**156.3**	256
*E. coli* ATCC 25922	625	312.5	**>625**	–

## Data Availability

The structure parameters of the obtained compound were deposited with the Cambridge Structural Database (CCDC 2584822) deposit@ccdc.cam.ac.uk or http://www.ccdc.cam.ac.uk/data_request/cif (accessed on 3 September 2026).

## Supplementary Materials

The following supporting information can be downloaded at https://www.mdpi.com/article/10.3390/molecules31203676/s1, Figure S1: IR-Spectra of **1** (solid sample, RT, attenuated total reflection technique); Figure S2: Step-by-step synthesis of complex **1**; Figure S3: Experimental (blue curve) and calculated (red curve) powder diffraction patterns for compound **1** and their difference (grey curve); Figure S4: Step-by-step synthetic operations of SnI_2_ preparation; Figure S5: Experimental (blue curve) and calculated (red curve) powder X-ray diffraction patterns of SnI_2_ and their difference (grey curve); Figure S6. ^1^H NMR of [(4-MeO-BIAN)SnI(μ-I)]_2_ (300 MHz, DMSO-d6).
