# Supplementary material for: Synthesis, Structure and Antibacterial Activity of a Neutral Tin(II) Iodide Complex with 1,2-bis[(4-Methoxyphenyl)imino]acenaphthene
Source: Molecules. 2026 Oct 14;31(20):3676. doi: 10.3390/molecules31203676 (PMC13707657; doi:10.3390/molecules31203676)
Supplement: Supplementary file 1 [file molecules-31-03676-s001.zip › molecules-4574284-supplementary.pdf]

molecules

Supplementary Materials

Synthesis, structure and antibacterial activity of a neutral tin(II) iodide complex with 1,2-bis[(4-methoxyphenyl)imino]acenaphthene

Anna A. Tychinina [1,2], Ivan V. Smolyaninov [1], Natalia V. Gogoleva [1], Anton N. Lukoyanov [2,3], Dmitriy S. Yambulatov [1,*], Mikhail A. Kiskin [1]

1 Kurnakov Institute of General and Inorganic Chemistry, Russian Academy of Sciences, 31 Leninsky prosp., 119991 Moscow, Russian Federation. Fax: +7 (495) 955 4817
2 D. Mendeleev University of Chemical Technology of Russia, 9 Miusskaya Square, 125047 Moscow, Russian Federation. Tel.: +7 (499) 978-86-60
3 Razuvaev Institute of Organometallic Chemistry, Russian Academy of Sciences, 49 Tropinina Street, 603137 Nizhny Novgorod, Russian Federation. Fax: +7 (831) 462-74-97
* Correspondence: yambulatov@igic.ras.ru or yambulatov@yandex.ru (D. S. Y.)

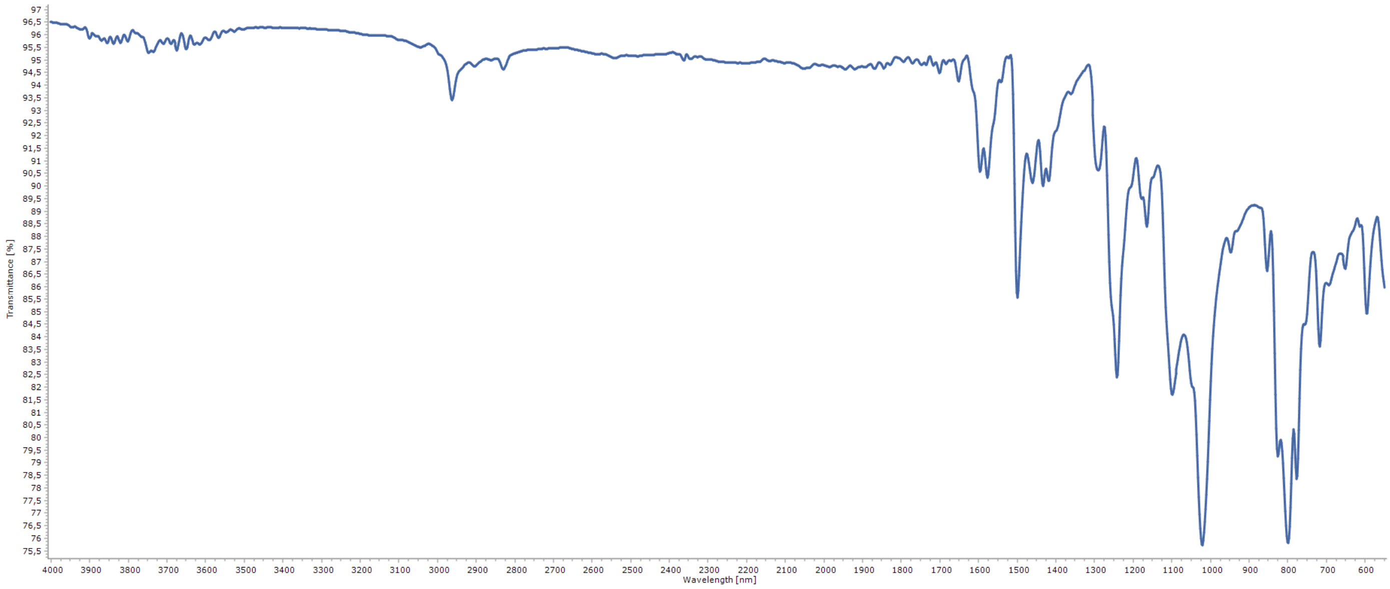

Figure S1. IR-Spectra of **1** (solid sample, RT, attenuated total reflection technique).

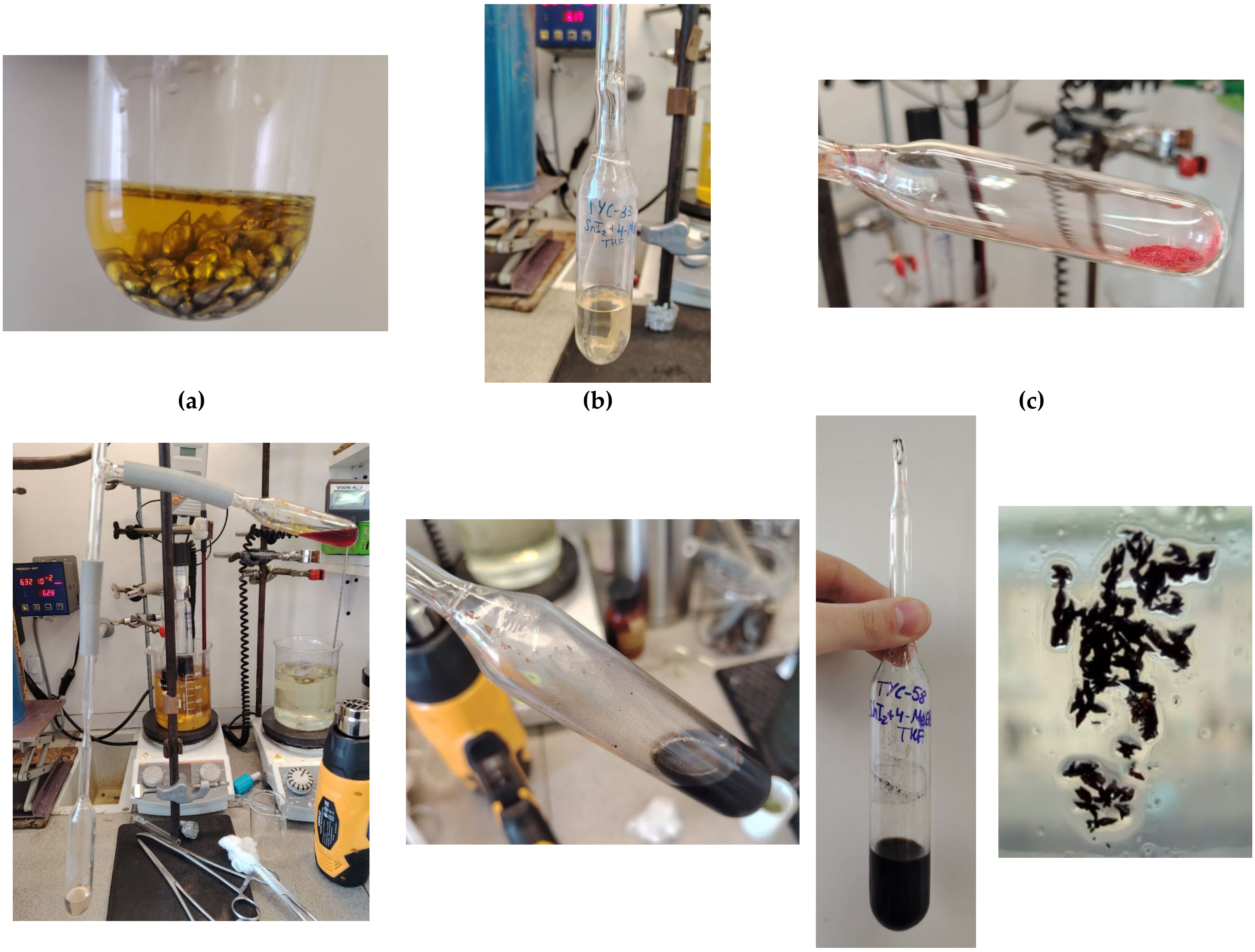

(a) **(b)** **(c)**

(d) **(e)** **(f)** **(g)**

Figure S2. Step-by-step synthesis of complex **1**: (a) a solution of SnI_2 in THF was prepared in a glass reactor with a Teflon stopcock; (b) the resulting SnI_2 solution was transferred into a glass ampoule; (c) the 4-MeO-BIAN ligand was placed in a separate glass ampoule; (d) anhydrous THF was condensed into the ampoule with the ligand, affording a ligand solution in THF; (e) the resulting ligand solution was transferred into the ampoule with the SnI_2 solution, the reaction mixture turned brown; (f) the ampoule was sealed and left to stir at room temperature; (g) keeping the reaction mixture at room temperature led to the formation of dark red crystals of compound **1** with a rhombic habit.

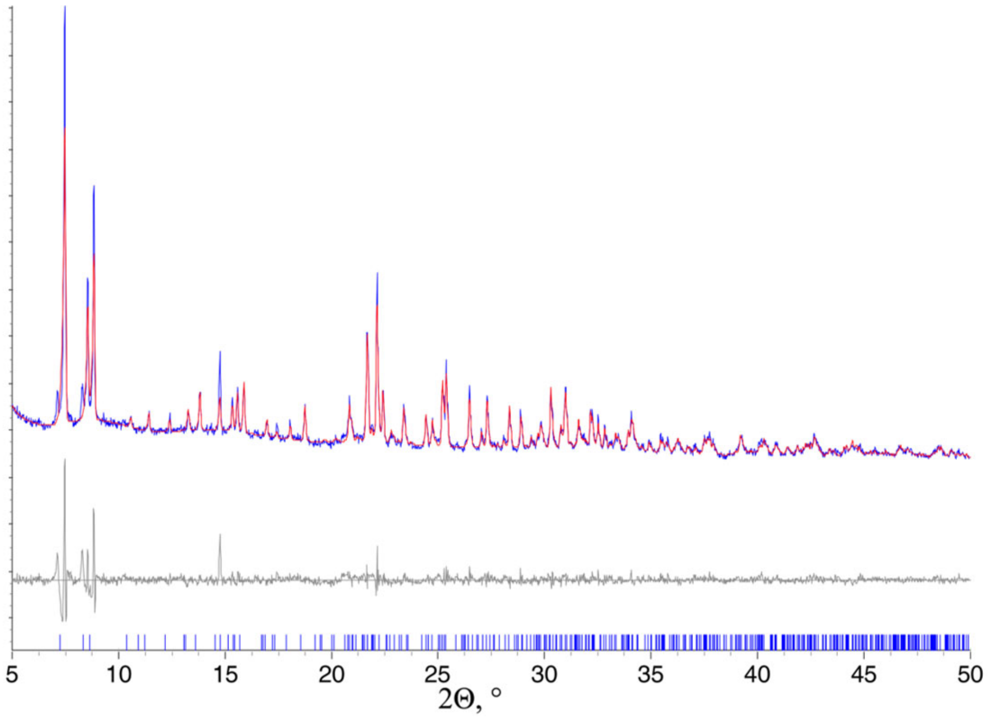

Figure S3. Experimental (blue curve) and calculated (red curve) powder diffraction patterns for compound **1**, and their difference (grey curve). Tick marks indicate the theoretical peak positions.

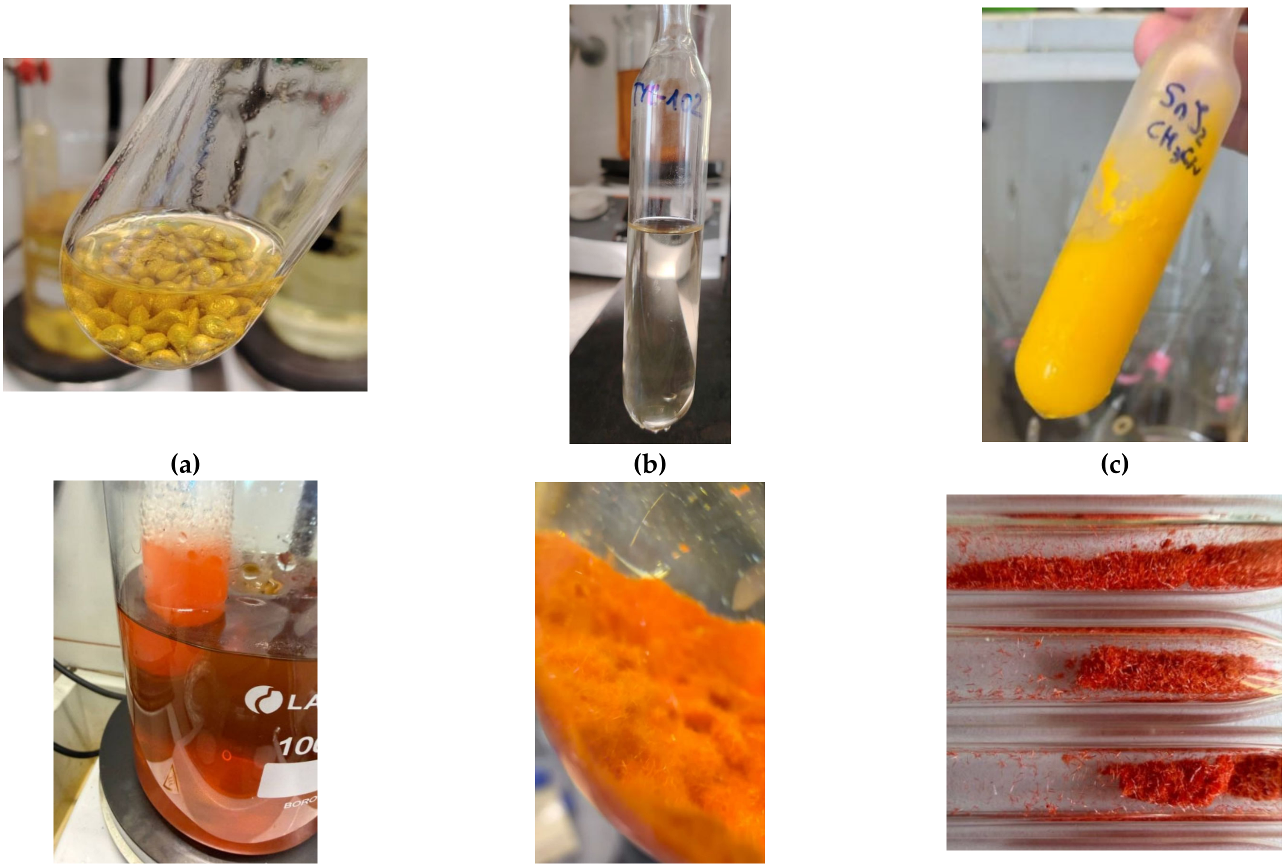

(a) (b) (c) (d) (e) (f)

Figure S4. A weighed amount of iodine (0.254 g, 1 mmol) was dissolved in anhydrous THF on a vacuum line. The resulting iodine solution was transferred onto excess of metallic tin (a). The resulting tin(II) iodide solution was then separated from the metallic tin (b). The THF was removed, and acetonitrile was condensed onto the resulting tin(II) iodide to afford an orange solution (c). The ampoule was sealed and heated in an oil bath at 90 °C (d), which led to the formation of orange needle-like crystals under the solvent layer (e). Upon isolation and transfer of the crystals into ampoules in the absence of solvent, the crystals turned red (f).

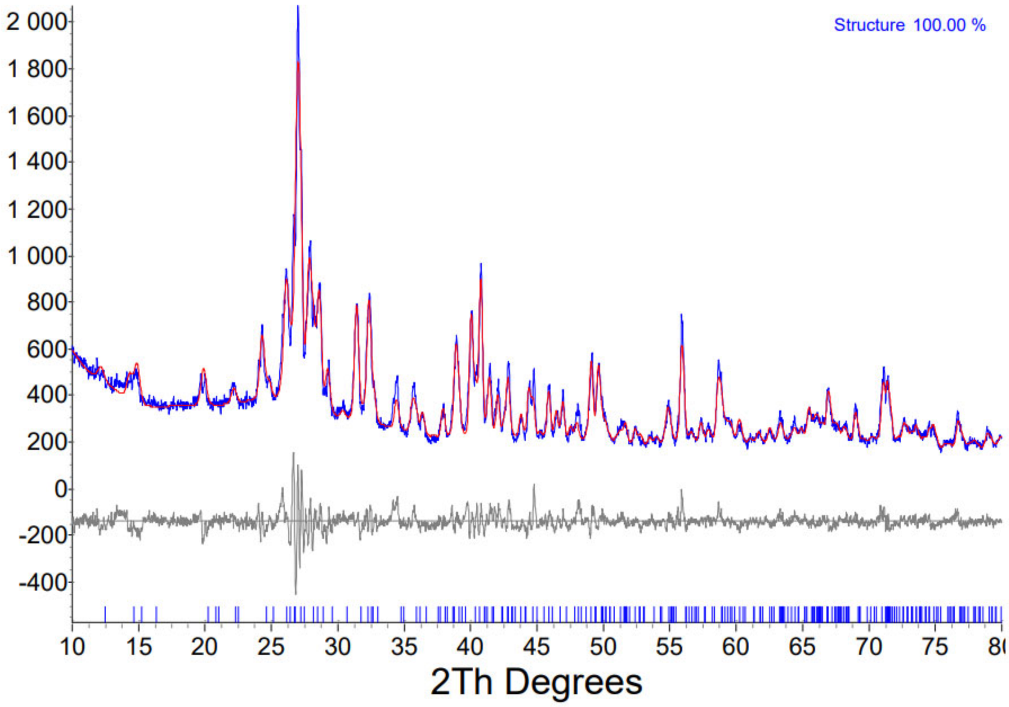

Figure S5. Experimental (blue curve) and calculated (red curve) powder X-ray diffraction patterns of SnI_2, and their difference (grey curve). The Rietveld refinement confirms that the obtained SnI_2 is a single-phase crystalline material.

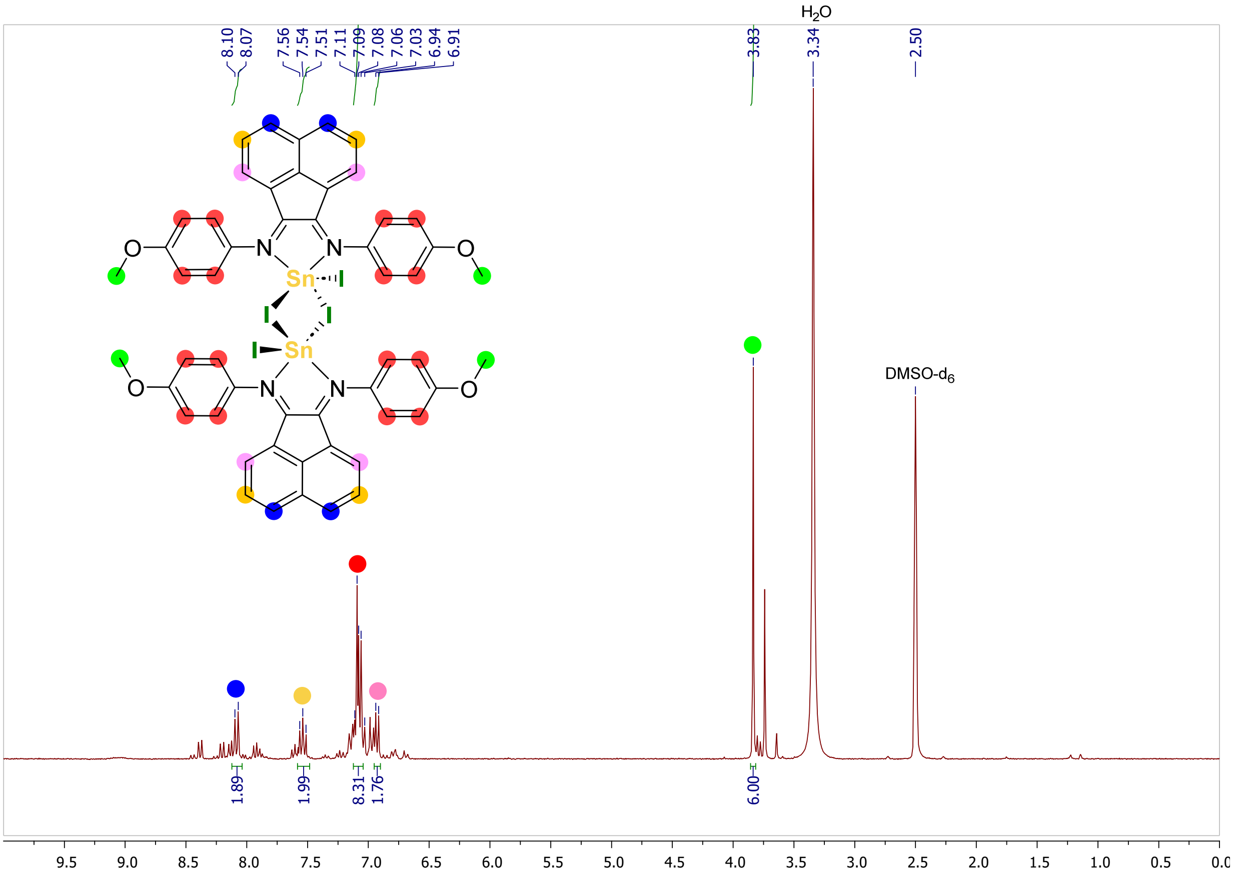

Figure S6. ^{1}H NMR of [(4-MeO-BIAN)SnI(μ-I)]$_2$ (300 MHz, DMSO-*d6*) δ, ppm: 8.09 (d, 2H, C(5)H, J = 8.2 Hz), 7.54 (t, 2H, C(4)H, J = 7.6 Hz), 7.11-7.03 (m, 8H, C_6H_4), 6.93 (d, 2H, C(3)H, J = 7.2 Hz), 3.83 (s, 6H, OMe)
